# Annotations

**Published:** 1908-01-18

**Authors:** 


### Annotations.

### Malaria in the Roman Campagna.

Dr. Luigi Egidi, who has taken an active part in the fight against malaria in the Roman Campagna, gives, in a recently issued pamphlet, an encouraging account of the progress which has been made towards the suppression of this disease. Since 1900 the Italian Red Cross Society has conducted an anti-malarial campaign from June to October, inclusive, in each year. For this purpose the whole of the Roman territory is divided into seven sections, and a sanitary station in charge of a doctor and a nurse is established in each section. All the stations are protected by wire screens and thin nettings, and the staff wear masks and gloves in order to avoid mosquito bites. A horsed ambulance is also provided, as well as one or more saddle horses, and in this way the surgeon and his assistants are able to cover the section placed in their charge. With a view to prophylaxis quinine is distributed for daily administration to all persons living in malarial districts, and this preventive measure is usually successful. Active treatment of infected persons is undertaken, and the patients are regularly visited in their homes, though very serious cases are removed to a hospital in Rome. As a result of these measures there has been an enormous reduction in the number of sufferers from malaria. In 1900 it was estimated that 31 per cent. of the population were affected by the disease, whereas in 1906 the numbers fell to 3.4 per cent. Another gratifying feature of the campaign is the decrease in the proportion of cases of a malignant type. These in 1900 numbered 27; in the following year there was a rapid decline; and in 1906 not a single instance was recorded. As Dr. Egidi says, "These figures need no comment."

### Regulation of Fees in the Indian Medical Service.

Some few months ago attention was directed to an order issued by the Government of India relative to the fees to be accepted by medical officers for professional attendance on ruling chiefs and other Indian gentlemen of high position. In this it was laid down that no fee beyond a certain specified sum must be demanded or accepted except with the permission of the Director-General of the Indian Medical Service. This regulation was to be applied whenever the professional charges were calculated on a scale in excess of 16 rupees, or in certain exceptional cases 32 rupees, per visit, or when the total amount for attendance during any one month exceeded 160 rupees. Naturally, those concerned with the interests of the Indian Medical Service had some very plain-spoken criticisms to pass on a rule which reflected in a very unpleasant fashion on the honour and good faith of a large body of the medical profession. It is satisfactory to find that an influential body of lay opinion in India endorses this position. From copies of journals recently to hand it is obvious that a very strong feeling has been aroused on the subject. In one of these it is urged that the medical officers in India have a record of which any body of men may be proud, that no other officials do so considerable an amount of gratuitous work, and that the new regulation treats the medical service as though its members were "potential extortioners." If any case of exorbitant charges occurs, by all means let the offender be suitably dealt with, but this is quite a different proposal from a rule which implies that all the members of an honourable service are to be regarded with suspicion. There is also another aspect to the question. The regulation not only applies compulsion to the medical officer, but also to the patient, and it would be strange indeed if an Indian gentleman approved of an order which prevented him expressing in practical fashion his appreciation of medical services rendered to himself or his family unless with the consent of the governing authorities. And this objection becomes all the stronger when it is remembered that to obtain such consent there must be presented a confidential report in which, necessarily, personal or family matters, sometimes of great delicacy, would be revealed.

### The "Albion Magazine."

This is a monthly journal published in the interests of the deaf. Moreover, it is the property of a firm of deaf men and women, is edited by a deaf editor, illustrated by deaf artists, and, for the most part, written by deaf contributors. It may, therefore, fairly claim to be a unique publication. As regards its utility, the claim is made that it will endeavour to rescue the deaf from the isolation to which, in greater or less measure, they are condemned, to suggest occupations and interests for them, and to discuss genuine remedies for deafness as well as to denounce the quacks who make the deaf their special prey. In the first number of the magazine considerable vigour is shown in the last-mentioned direction. Various "new" and "original" and "wonderful" aids for the deaf are examined and explained, and their value and limitations are duly set forth. Inventions of this order are recognised as periodical events, and in all such devices there is found to be a close family resemblance. Still further, readers of the journal are advised not to send money to a number of specified "aural quacks" whose advertisements appear more or less prominently in the lay press. Concerning these there is no lack of plain speaking. Thus, of one advertiser it is said "he is a frowsy little fraud and his treatment a worthless humbug"; another, who styles himself a "Professor," uses testimonials "which show certain signs of manipulation and is most decidedly to be shunned"; a third is dubbed "the Prince of Fakirs"; and a general warning is given against all "Institutes" which profess to cure deafness. In all this there is a wholesome frankness which is much to be commended, and which can hardly fail to protect at least some individuals from the clutches of unscrupulous quacks. Another useful feature of the magazine is a list of institutions where deaf children are received and trained. The *Albion Magazine* has made a promising start, and we wish it a useful and successful career.

